# Decomposition Analysis of High BMI‐Attributable Stroke Burden in China: Population Aging as the Dominant Driver

**DOI:** 10.1155/jobe/6789331

**Published:** 2026-09-30

**Authors:** Xiaoxue Liu, Haoyun Zhou, Runtang Meng, Chuanhua Yu, Fang Wang, Yongchao Liu

**Affiliations:** ^1^ Department of Epidemiology and Biostatistics Wuxi School of Medicine Jiangnan University Wuxi Jiangsu 214122 China jiangnan.edu.cn; ^2^ Department of Preventive Medicine School of Public Health and Nursing Hangzhou Normal University Hangzhou Zhejiang 311121 China hznu.edu.cn; ^3^ Department of Epidemiology and Biostatistics School of Public Health Wuhan University Wuhan Hubei 430071 China whu.edu.cn; ^4^ Department of Epidemiology and Biostatistics School of Public Health Xuzhou Medical University Xuzhou Jiangsu 221004 China xzmc.edu.cn; ^5^ Department of Chronic Non-Communicable Disease Control Affiliated Wuxi Center for Disease Control and Prevention of Nanjing Medical University Wuxi Center for Disease Control and Prevention Wuxi China

**Keywords:** DALY, high BMI, mortality, stroke

## Abstract

**Background:**

High body‐mass index (BMI) is the second largest contributor to stroke burden and the fastest‐growing risk factor for stroke globally. Given China’s rapidly aging population and the increasing obesity epidemic, understanding the long‐term trends and drivers of stroke burden is critical.

**Objectives:**

High BMI is a chief risk factor for stroke. We aim to evaluate the long‐term trends and population risk on stroke mortality burden caused by high BMI in Chinese population between 1990 and 2023.

**Methods:**

The decomposition analysis and linear regression were utilized to determine the contribution of population aging and other risk factors to the changing trends in the mortality and disability‐adjusted life year (DALYs) of stroke caused by high BMI.

**Results:**

In China, the mortality rate of stroke caused by high BMI increased by 1.640/100,000 during the study period of 1990–2023. The increase was decomposed as 1.180/100,000 due to population aging and 0.460/100,000 due to other risk factors, with population aging contributing 72% and other risk factors contributing 28%. The DALYs rate increased by 52.228/100,000, of which 32.999/100,000 was due to population aging and 19.229/100,000 was due to other risk factors. Notably, population aging contributed 63% of the increase. These findings confirm that population aging was the dominant driver of the increased stroke burden attributable to high BMI, whether measured by mortality or DALY.

**Conclusions:**

Population aging is the dominant driver of the increasing stroke burden attributable to high BMI in China. Effective burden reduction will require integrated prevention strategies targeting both the elderly population and modifiable metabolic risk factors.

## 1. Introduction

High‐body mass index (BMI) has been at the top risk factors contributing to the global disease burden [1–5]. As reported, the proportion of global adults with a BMI of 25 kg/m(2) or greater increased from 28.8% to 36.9% in men and from 29.8% to 38.0% in women between 1980 and 2013 [4]. It is widely known that BMI trajectories over age and time are highly variable across countries [6, 7]. The prevalence of adult obesity in the United States increased from 30.5% in 2000 to 42.4% in 2018 [8]. The prevalence of overweight and obesity among Chinese adults aged 18–69 years was 34.4% and 16.8% in 2018, respectively [9]. Besides, Chinese children and adolescents also face the problem of obesity [10]. Since 2010, there have been sizeable increases in disability‐adjusted life years (DALYs) attributable to metabolic risk factors and noncommunicable diseases in growing and aging populations [2].

High BMI is considered a chief risk factor for stroke [9, 11–13]. It is the second largest contributor to the stroke burden and the fastest‐growing risk factor for stroke between 1990 and 2019 [14]. In China, stroke is the leading cause of DALYs, with the number of stroke DALYs reaching 53.2 million in 2021 [15]. Given both increasing exposure to obesity and large population aging in China, it is crucial to evaluate trends and main driving factors on mortality and DALYs rates of stroke.

## 2. Materials and Methods

### 2.1. Data Sources

Mortality and DALYs data of stroke were obtained from the GBD dataset (https://ghdx.healthdata.org/gbd-results-tool). The original data of stroke mortality and DALYs were nationally representative [2]. Stroke is defined according to World Health Organization (WHO) criteria as rapidly developing clinical signs of focal (or less commonly global) disturbance of cerebral function lasting more than 24 h or leading to death with no apparent cause other than that of vascular origin [14]. Ethics approval and patient consent to participate are not applicable in this study.

In this study, the exposure level associated with minimum risk, called the theoretical minimum risk exposure level, for high BMI is between 20 and 25 kg/m(2). The mortality rate of stroke attributable to high BMI was estimated based on defining population attributable fraction (PAF) through combining distribution of exposure to BMI with exposure‐risk estimates at each level of exposure. Attributable deaths and DALYs cases were computed by multiplying PAFs by the relevant outcome quantity for each age‐sex‐location‐year.

### 2.2. Statistical Analysis

We used linear regression to evaluate annual percentage change (APC) in the mortality and DALYs rates of stroke attributable to high BMI over years. In the linear regression, the natural logarithm of the mortality or DALYs rate was used as the dependent variable and the year as the independent variable. We computed the APC by age–sex using the following general formula: APC = 100 × (e^*β*^ − 1); *β* represents the estimated coefficient from the linear regression.

The sharp rise in chronic disease mortality rates can generally be attributed to two factors: first, the aging of the population structure, and second, the influence of other risk factors. Nondemographic factors refer to the sum of all factors other than demographic factors that may lead to changes in mortality rates, including genetic, environmental, and behavioral factors. The mortality differential decomposition method is a widely used method in demographic studies to answer the question, “How much of the difference in mortality between population A and population B is due to the age difference between the two population?” Therefore, decomposition analysis can be used when analyzing mortality data for two areas or two generations [16–18]. For two generations in the same region, we can determine how much of the change in mortality between the two generations is due to population aging and how much is due to changes in other risk factors by decomposition analysis method. The decomposition approach could incorporate interactions among component effects, such as population growth, population aging, and mortality pattern transitions, into the additive main effects, reported in previous studies [16, 17]. To decompose the difference between the crude mortality rates of population A and population B, we considered CDR^B^ as the crude mortality rate of population B, CDR^A^ as the crude mortality rate of population A, and *diff* as the difference between the mortality rates of the two population. *C* presents the age composition of the populations, and *M* presents the age‐specific mortality rate; we follow the following formula:

(1) diff=CDRB−CDRA=∑CXBMXB−∑CXAMXA.

We further summarized the difference between the mortality rates of the two population and the difference in age composition of the two populations as follows:

(2) diff=∑CXB−CXA MXB+MXA2+∑MXB−MXA CXB+CXA2.

= Difference in age structure (weighted by mean of age‐specific mortality rates of population A and population B) + Difference in mortality rates (weighted by age structure of population A and population B).

= Impacts of age structure difference + Contribution percent of mortality difference impacts.

In our decomposition framework, “nondemographic factors” refers to the sum of all factors other than changes in age structure that may lead to changes in mortality or DALY rates. These encompass, but are not limited to, changes in the prevalence and intensity of high BMI exposure; improvements in medical care and stroke treatment effectiveness; changes in healthcare access and quality; lifestyle and behavioral modifications (e.g., diet, physical activity, and smoking); and broader socioeconomic developments that influence stroke risk and outcomes. Importantly, our decomposition approach does not attribute the “nondemographic” component to any specific factor—rather, it represents the collective residual effect of all age‐independent changes.

Specifically, the original GBD 1990 estimates for the 20–24 and 25–29 age groups yielded negative mortality rates, which are biologically implausible. This issue likely arises from statistical smoothing or modeling artifacts in the GBD estimation framework, particularly in strata with very small numbers of events. To ensure the validity of our results, we set these negative values to zero in decomposition analysis. The original negative values of mortality were very close to zero in magnitude, and these two age groups represent only a negligible proportion of the total stroke burden. Therefore, this adjustment has no material impact on the overall findings, trends, or conclusions of our study. All analyses were carried out with Stata 12.0 software (StataCorp, College Station, TX, USA).

## 3. Results

The crude rates of stroke death and DALYs caused by high BMI have been increasing in Chinese population during 1990–2023 (Figure 1). The crude rates of them annually increased by 2.757% and 2.708% (*p* < 0.001), respectively (Table 1). Compared with the global level, trends in the age‐standardized mortality and DALYs rates related to high BMI tended to be stable in China (Figure 2). We also found gender difference that the rate significantly increased in males (Figure 1).

**FIGURE 1:**
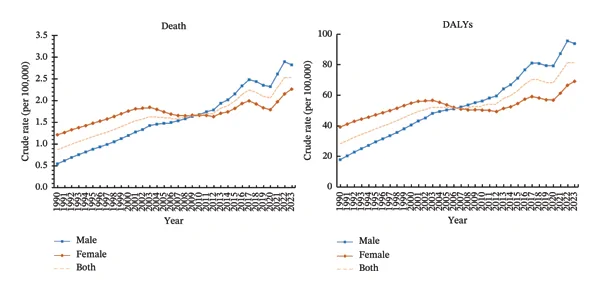
The crude rate of stroke death and DALYs caused by high BMI in Chinese population during 1990–2023.

**TABLE 1:** Annual percent changes in death and DALYs rates of stroke caused by high BMI during 1990–2023.

Stroke	APC^∗^ (%)	
	Crude rates	Age‐standard rates
China		
Death	−0.880	−3.855
DALYs	−1.278	−3.780
Death: high BMI	2.757	0.390
DALYs: high BMI	2.708	0.581
Global		
Death	−0.799	−2.362
DALYs	−0.895	−2.145
Death: high BMI	0.288	−1.188
DALYs: high BMI	0.696	−0.545

^∗^All *p* < 0.001.

**FIGURE 2:**
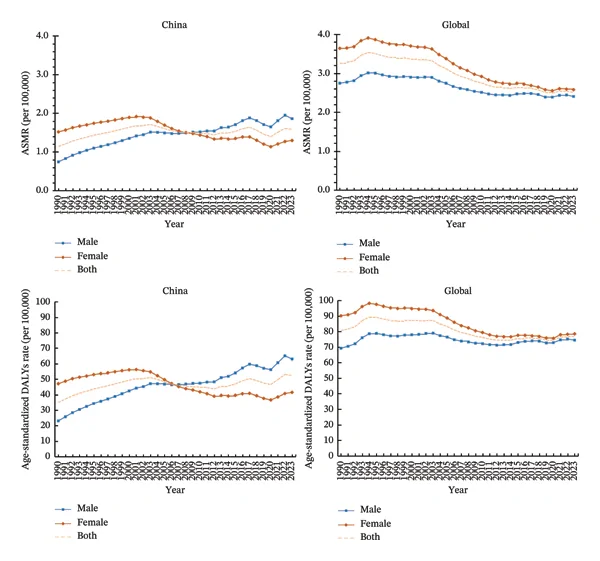
The ASMR and age‐standardized DALYs rates of stroke caused by high BMI in Chinese population, compared with global population.

### 3.1. Decomposition Analysis Results

Considering the effect of both risk factors and huge population aging, we conducted decomposition analysis to determine their contribution to the change in stroke burden. Table 2 shows the specific calculation of decomposition results. Table 3 shows the decomposition of changes in stroke mortality and DALYs caused by high BMI in China.

**TABLE 2:** Decomposition of changes in stroke mortality caused by high BMI in Chinese population between 1990 and 2023.

Age groups	Age structure of the population in 1990 $C_{X}^{A}$ *(1)*	Age‐specific mortality rate in 1990 $M_{X}^{A}$ *(2)*	Age structure of the population in 2023 $C_{X}^{B}$ *(3)*	Age‐specific mortality rate in 2023 $M_{X}^{B}$ *(4)*	Difference in population weight by age $C_{X}^{B}-C_{X}^{A}$ *(5)*	Weight *(* $M_{X}^{B}{+M}_{X}^{A}$ *)/2 (6)*	Difference in age structure of the population *(7) = (6)* × *(5)*	Difference in age‐specific mortality rate between two populations $M_{X}^{B}{-M}_{X}^{A}$ *(8)*	Weight *(* $C_{X}^{B}+C_{X}^{A}$ *)/2 (9)*	Difference in mortality rate *(10) = (8)* × *(9)*
20∼	0.102	0	0.0497	0.012644869	−0.0523	0.006322435	−0.0003	0.012644869	0.07585	0.000959113
25∼	0.096	0	0.0569	0.072114812	−0.0391	0.036057406	−0.001	0.072114812	0.07645	0.005513177
30∼	0.08	0.172091988	0.0764	0.222116691	−0.0036	0.19710434	−0.001	0.050024703	0.0782	0.003911932
35∼	0.075	0.360904001	0.0799	0.564780194	0.0049	0.462842097	0.002	0.203876192	0.07745	0.015790211
40∼	0.059	0.454956162	0.0683	1.049678794	0.0093	0.752317478	0.007	0.594722633	0.06365	0.037854096
45∼	0.047	0.446608098	0.0691	1.527122314	0.0221	0.986865206	0.022	1.080514217	0.05805	0.06272385
50∼	0.043	1.609726938	0.0854	2.396626682	0.0424	2.00317681	0.085	0.786899744	0.0642	0.050518964
55∼	0.04	3.347327196	0.0816	3.27507787	0.0416	3.311202533	0.138	−0.072249326	0.0608	−0.004392759
60∼	0.032	5.979657106	0.057	5.59355575	0.025	5.786606428	0.145	−0.386101355	0.0445	−0.01718151
65∼	0.025	8.705315747	0.055	8.275878874	0.03	8.490597311	0.255	−0.429436873	0.04	−0.017177475
70∼	0.017	7.852803798	0.0435	10.05728945	0.0265	8.955046623	0.237	2.204485649	0.03025	0.066685691
75∼	0.011	6.833980771	0.0266	11.24936393	0.0156	9.041672352	0.141	4.415383163	0.0188	0.083009203
80∼	0.006	4.866483231	0.0159	12.36416231	0.0099	8.615322771	0.085	7.49767908	0.01095	0.082099586
85∼	0.003	2.234091571	0.0091	13.60382949	0.0061	7.918960528	0.048	11.36973791	0.00605	0.068786914
90∼	0.0005	0.627821676	0.0033	11.47117731	0.0028	6.049499492	0.017	10.84335563	0.0019	0.020602376
总计 $\sum C_{X}^{A}M_{X}^{A}$ = 0.945 $\sum C_{X}^{B}M_{X}^{B}$ = 2.585			1.180					0.460		

**TABLE 3:** Decomposition analysis of differences in stroke mortality and DALYs rates caused by high BMI in China.

	1990	2023	Difference in rate (/100,000)	Impact of demographic composition (%)	Impact of other risk factors (%)
	Crude rate (/100,000)	Crude rate (/100,000)			
Mortality	0.945	2.585	1.640	1.180/100,000 (72%)	0.460 (/100,000) (28%)
DALYs	30.589	82.817	52.228	32.999 (/100,000) (63%)	19.229 (/100,000) (37%)

The increase in stroke mortality caused by high BMI was the result of the combined effect of demographic and nondemographic factors. The difference in crude mortality of stroke caused by high BMI between 1990 and 2023 was 1.640/100,000, of which 1.180/100,000 was due to the difference in age structure of the two populations and 0.460/100,000 was due to the difference in true mortality. In other words, demographic factors played a greater role than nondemographic factors. Population aging accounted for 72% of the increase and other risk factors explained 28%.

The increase in the DALYs rate was also influenced by both demographic factors and nondemographic factors (Table 3). The DALYs rate increased by 52.228/100,000, of which 32.999/100,000 was due to population aging and 19.229/100,000 was due to other risk factors. This demonstrates that population aging contributed 63% of the increase and other factors contributed 37%.

These findings confirm that population aging was the dominant driver of the increased stroke burden attributable to high BMI, whether measured by mortality or DALY.

## 4. Discussion

In this study, under the interaction of demographic and nondemographic factors, the mortality and DALYs rates of stroke related to high BMI in Chinese population increased from the 1990s to the 2020s, especially a significant increase in males. Previous study also observed an increase in stroke mortality over the past decades [19]. The increase in stroke mortality caused by high BMI was decomposed into a major component due to population aging (72%) and a minor component due to other risk factors (28%). The finding that population aging contributed approximately 72% of the increase in stroke mortality attributable to high BMI aligns with China’s well‐documented demographic transition. This suggests that, in the absence of population aging, the increasing burden of high BMI‐related stroke would have been substantially smaller. The nondemographic component (28% of the increase in mortality rate) represents the net effect of all age‐independent changes. This could include improved acute stroke care and risk factor management, which would have a decreasing effect, alongside increased high‐BMI prevalence and behavioral risk factors, which would have an increasing effect. The positive net value (0.460/100,000) suggests that, on balance, age‐independent risk factors (particularly rising obesity prevalence) may have outweighed improvements in stroke care and prevention over the study period. However, we caution that this residual component is not directly attributable to any single factor.

Previous studies showed aging in upper‐to‐middle income countries and population growth in low‐to‐middle income countries further offset endeavors to reduce the deaths of ischemic stroke [20]. In our study, the increase in the DALYs rate of stroke caused by high BMI was decomposed as population aging (63%) and other risk factors (37%). This result also indicated population aging contributed to the majority of the increase in the DALYs rates stroke caused by high BMI between 1990 and 2023 populations and other risk factors contributed to a small portion of the increase. Consistent with our research findings, previous study also indicated that for older age groups, metabolic risks such as high SBP, high BMI, and high FPG have a greater impact on disease burden and a 49.4% increase in DALYs caused by metabolic risks, all reflecting aging populations and changing lifestyles on a global scale [21]. It also suggested age‐standardized global DALY rates increased considerably due to both increasing risk exposure and population aging (e.g., high BMI) [21]. Currently, there is still a lack of relevant research on the decomposition analysis of stroke burden in China. Previous study reported that global ischemic stroke attributed to high low‐density lipoprotein cholesterol (IS‐hLDL‐C) with a decomposition analysis [20], which found variation in territories with high to middle high SDI played an important role in the change.

Therefore, the decomposition analysis in our study suggested that the contribution of population aging to the increase in the mortality and DALYs rates of stroke caused by high BMI was relatively higher in China over the long‐term study period. As China faces a huge population aging and the rapid development of urbanization and migration, it is urgent and necessary to improve lifestyle and behavioral patterns related to metabolic factors for the control of stroke burden.

Besides, this study found the crude mortality and DALYs rates of stroke caused by high BMI increased in males but decreased in females during the same period. This increase may be explained by an increase in exposure to the risk factors related to stroke [22–24]. The difference across gender was possibly related to the rise in mean level of BMI among the adults and the divergent trends in BMI by sex over the past decades [22]. As reported, 85 million adults aged 18–69 years in China were obese in 2018, which was three times as many as those in 2004 [22]. Previous studies showed stroke was also related to blood pressure management, diabetes, coronary artery disease, and changes in dietary structure, people’s behavior, and lifestyle [25, 26]. While lifestyle factors that have been proven to reduce risk of stroke included reducing salt, eliminating smoking, performing regular physical activity, and maintaining a normal body weight [27–29]. The recent emerging risk factors for stroke, such as obstructive sleep apnea and electronic cigarettes, could be discussed in the future [25].

### 4.1. Limitations

There are some limitations. First, we acknowledge that the uncertainty intervals for the GBD mortality estimates used in our decomposition analysis were relatively wide for some age groups, reflecting the inherent uncertainty in the GBD modeling framework. However, the Monte Carlo simulation demonstrated that despite this uncertainty, the conclusion that population aging is the dominant driver of the observed increase remained robust. Second, we lacked survey data on the risk factors and were unable to provide and discuss the prevalence of high BMI in the Chinese population. Third, the decomposition analysis quantifies the relative contributions of demographic aging and all other factors combined to the observed changes in stroke burden. It does not establish causal relationships between specific nondemographic factors and the observed trends. The other risk factors component should be interpreted as a composite residual that warrants further investigation using individual‐level data and targeted study designs. Fourth, the findings from this decomposition analysis should be interpreted within the context of the GBD modeling framework. The GBD 2023 estimates represent the best available synthetic evidence, but they rely on heterogeneous data sources, modeling assumptions, and imputations for countries and regions with limited primary data. Our decomposition builds upon these model‐derived outputs and should, therefore, be considered as providing directional and relative insights rather than precise causal estimates. However, we acknowledge that the decomposition approach assumes that the GBD estimates accurately reflect the true underlying disease burden and risk factor exposures, which may not fully capture all real‐world complexities. Future studies using primary data collection with longitudinal follow‐up would strengthen the evidence base for the trends observed.

## 5. Conclusions

The mortality and DALYs rates of stroke related to high BMI have been increasing since 1990. However, population aging is the dominant driver of the increasing stroke burden attributable to high BMI in China. Effective burden reduction will require integrated prevention strategies that address both the aging population structure and modifiable metabolic risk factors.

## Author Contributions

Xiaoxue Liu: conceptualization, methodology, software, data curation, and writing–original draft preparation. Haoyun Zhou: supervision. Runtang Meng: writing–reviewing and editing. Chuanhua Yu: writing–reviewing and editing. Fang Wang: writing–reviewing and editing. Yongchao Liu: writing–reviewing and editing.

## Funding

This work was funded by the Basic Research Program of Jiangsu (grant number BK20241622), National Natural Science Foundation of China (grant number 82404375), and Wuxi Science and Technology Development Fund Project (grant number K20231046).

## Ethics Statement

The datasets analyzed during the current study are published and available in the Global Burden of Disease database repository. Thus, ethics approval and patient consent to participate were not applicable in this study.

## Conflicts of Interest

The authors declare no conflicts of interest.

## Data Availability

The datasets [2] analyzed in the current study are available in the GBD repository, https://ghdx.healthdata.org/gbd-results-tool.
